# Surgical Trainee Opinions in the United Kingdom Regarding a Three-Dimensional Virtual Mentoring Environment (MentorSL) in Second Life: Pilot Study

**DOI:** 10.2196/games.2822

**Published:** 2013-09-20

**Authors:** Usman Jaffer, Nigel W John, Nigel Standfield

**Affiliations:** ^1^Imperial College Healthcare Trust, Hammersmith HospitalLondonUnited Kingdom; ^2^School of Computer ScienceUniversity of BangorBangorUnited Kingdom

**Keywords:** education, Internet, training

## Abstract

**Background:**

Medical mentoring is becoming increasingly complex with the evolving needs of trainees and the complexities of their personal and social lives. The Internet is an enabling technology, which increasingly facilitates interaction with multiple people at a distance. Web 2.0 and 3.0 technology shows promise in furthering this facilitation.

**Objective:**

The objective of our study was to establish opinions among doctors in postgraduate surgical training regarding mentoring and whether these doctors would readily accept virtual mentoring following a brief experience.

**Methods:**

On the 12th of February 2012, an introductory teaching class was arranged by The London Postgraduate School of Surgery for doctors in training. Participants were introduced to a novel virtual mentoring system and asked to complete a questionnaire regarding their opinions before and after the demonstration.

**Results:**

A total of 57 junior doctors attended. Among them, 35 completed questionnaires pre- and postdemonstration. Regarding usefulness of a 3D virtual environment for mentoring, 6/35 (17%) agreed or strongly agreed and 20/35 (57%) were unsure prior to the session. Following 20 minutes using MentorSL, this significantly increased to 14/35 (40%) agreeing or strongly agreeing with 11/35 (31%) unsure (*P*<.001). Prior to using MentorSL, regarding usefulness of voice communication for virtual mentoring, 11/35 (31%) agreed or strongly agreed and 18/35 (51%) were unsure. Following 20 minutes using MentorSL, 19/35 (54%) agreed or strongly agreed and 10/35 (29%) were unsure of usefulness. Regarding ease of use of navigation, search mentor, meeting scheduling, and voice communication features, 17/35 (49%), 13/35 (37%), 15/35 (43%), and 16/35 (46%) participants agreed or strongly agreed, respectively. Regarding usefulness of telementoring, 24/35 (69%) agreed or strongly agreed, increasing to 28/35 (80%) following the introduction. For usefulness of multiple mentors, initially 24/35 (69%) agreed or strongly agreed increasing to 29/35 (83%). For overall satisfaction, 30/35 (86%) reported good or adequate and 19/35 (54%) agreed or strongly agreed with using the system again.

**Conclusions:**

These data suggest that a short introduction on how to use virtual systems may result in significant participation and use of virtual mentoring systems.

## Introduction

### Background

Doctors in postgraduate surgical training often require guidance to overcome hurdles associated with modern-day surgical training. Good mentoring delivered in a timely fashion is a way in which surgical trainees may be helped through these difficulties in a manner compatible with the principles of adult learning.

The Standing Conference on Postgraduate Medical and Dental Education (SCOPME) in the United Kingdom, describes mentoring as:

The process whereby an experienced, highly regarded, empathic person (the mentor) guides another individual (the mentee) in the development and re-examination of their own ideas, learning, and personal and professional development. The mentor, who may or may not work in the same organization or field as the mentee, achieves this by listening and talking in confidence to the mentee [1]

The mentors have many roles that have previously been reviewed [2]. Briefly, these include advisor, coach, counselor/guide, and role model. As someone who has successfully negotiated some of these difficulties, a mentor may offer motivation, hope, and advice for the mentee. As technology increasingly becomes part of a managed learning process, expert mentoring of trainees, facilitated by technology, may become essential for ensuring patient safety.

It has been previously reported that trainees often do not have mentors or are unaware of the role of the mentor and therefore do not have beneficial meetings with them [2,3]. The management concept “Just-in-time” was popularized by the Toyota Motor Company and resulted in huge increases in efficiency and productivity. The essence of the system is to respond to needs and only call upon resources when they are required [4]. A parallel may be drawn with mentoring in that it is potentially a labor-intensive and costly resource, which is not necessarily required at all times. Mentoring may be best achieved in a “just-in-time” fashion where an appropriate mentor is available to facilitate problem solving in response to a real-world need. In order for this mentoring to be achieved in a comfortable learning environment, a knowledgeable, yet not necessarily proximate mentor may be most suitable.

### World Wide Web

The latest digital technologies may be a key enabler to support these requirements. The Department of Health in the United Kingdom has recently published a “Framework for Technology Enhanced Learning” that advocates the use of e-learning and simulation to enhance learning where there is a clear benefit to patient care [5]. Internet-based technology developments, including the World Wide Web (WWW), allow for increasing interactivity and may be of use in fulfilling tele- and multiple-mentoring needs. Improved mentoring may lead to improved trainee development, which may lead to improved patient care. There has been an evolution of the ways that interaction is facilitated and information processed and retrieved in the WWW [6]. Today’s WWW provides for an immersive, interactive, and information-rich potential resource. e-mentoring has been shown to be efficacious in the context of North American school children, interestingly reporting that the frequency of mentor-mentee interaction moderates the relationship between mentee “self-efficacy” and previous Internet experience with positive outcome [7]. The Web 3.0 format encompasses virtual worlds, the semantic Web, microformats, natural language search, data mining, machine learning, recommendation agents, artificial intelligence, and augmented reality technologies. Augmented reality involves a fusion of the physical world and computer-generated content, potentially delivered through the Internet. The use of augmented reality for anatomy education has been demonstrated [8], and there is great potential for this technology.

### MentorSL

Virtual worlds including Second Life (SL) by Linden Laboratories [9] and Olive by Forterra Systems [10] provide content as a three-dimensional (3D) environment in which we can navigate and interact with others as virtual representations of ourselves, avatars. Second Life, currently the most popular virtual world, facilitates streaming audio/video/TV/YouTube collections, 3D virtual libraries, and virtual tourist attractions and destinations [11]. A virtual emergency department training study has reported that ease of use and limited access to the software were identified as barriers to adoption [12]. Meskó summarized educational applications’ uses of SL [13]. He discussed potential advantages as being global collaboration without boundaries; interactivity in a manner better than a videoconference with use of videos, presentations, images, and Web links at the same time in one place. Being able to draw from a worldwide pool of experts and having the ability to establish exhibits which are not possible via a videoconference [14] or a website are also cited as advantages [15,16]. Interactions between SL residents may benefit each others’ participation via networks that allow for dynamic, evolving systems all made possible by “semantic” Web technology [17]. Virtual reality resources have been successfully used as educational resources [18-27].

**Figure 1:**
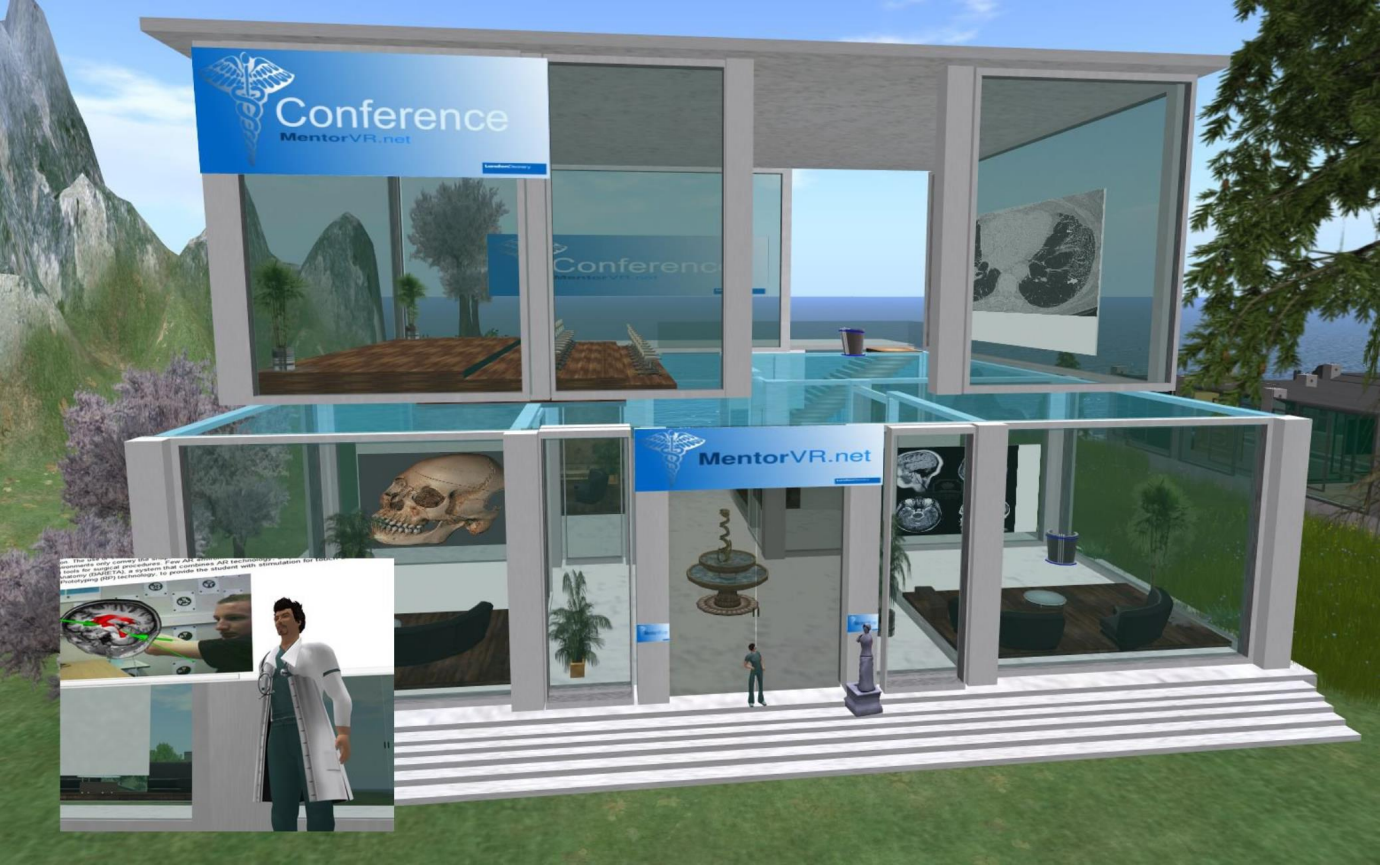
Photograph from Second Life showing the MentorSL meeting complex. Licensed under Creative Commons Attribution 2.0.

For the purposes of exploring new methods to support mentoring, a 3D virtual system, MentorSL, was developed [28]. Avatars assemble in a registration area, where they have a range of mentoring databases available to them (Figure 1). Mentors are able to log on to SL as avatars and provide mentoring, through virtual world communication, to their mentees. A range of online mentoring resources is available to both mentors and mentees and there are links to other mentoring resources.

Doctors in postgraduate surgical training in the London Postgraduate School of Surgery (see Textbox 1) were invited to experience the system and submit their views. The primary aims of our study were to establish whether barriers existed to the adoption of the 3D virtual mentoring environment and to establish whether a short introduction would be sufficient to achieve participant “buy-in”. The secondary aims were to establish which aspects of the system were deemed most useful and which further aspects should be developed further.

**Doctors in postgraduate surgical training.**

The London Postgraduate School of Surgery is the largest surgical training organization in the world. It is responsible for managing more than 900 trainees. The school offers programs at prestigious teaching centers across the capital city. Doctors in postgraduate training spend an initial 2 years (FY1, FY2) in generic foundation training; this is followed by a further 2 years (CT1, CT2) in core surgical training. Successful competitive progression results in spending an additional 6 years in specialty surgical training (ST3 to ST8) toward award of completion of training.

## Methods

### Study Participants

Doctors in postgraduate surgical training were invited to attend a “taster” session introducing virtual mentoring via the online virtual world SL at the London Postgraduate School of Surgery, UK. This session was a subsection of a wider training meeting being held. The trainees were within the first four years of postgraduate training. All participants had previously experience of mentoring as a component of “Foundation Training” which includes appointing of an “Assigned Educational Supervisor” by the training program. A 10-minute presentation on SL and specifically on MentorSL including an “in-world” walk through was given via a large screen projector. SL as a virtual world facilitating interaction of virtual people or avatars was explained. Methods used to navigating and communicate in SL were explained to participants. MentorSL was introduced as a tool to facilitate mentoring in the virtual world of SL. The search mentor facilities in MentorSL and the facilities to arrange and hold meeting within the MentorSL framework were explained and demonstrated. Following a short questions and answers session, participants were able to sit in groups at computer stations running Second Life fitted with multiple headsets. Facilitators in the real world as well as SL were available to help and guide participants.

### Data Collection

Participants were invited to fill in an anonymous questionnaire regarding their perceptions both prior to and after the session. The questionnaire consisted of 7 domains: (A) demographic data, (B) perceptions regarding mentoring, (C) perceptions regarding the 3D Web, (D) perceptions regarding the practicalities of MentorSL, (E) perceptions regarding tele- and multiple mentoring, (F) perceptions regarding further enhancements in virtual mentoring, and (G) perceptions regarding future use of MentorSL (see Multimedia Appendix 1 for the questionnaire). These questions were determined with a view to establishing whether doctors in postgraduate surgical training would readily accept use of a virtual mentoring facility and whether any particular aspect of the facility was related to future use of the system. Demographic data were only collected once, questions in domains (B), (C), and (E) were posed both before and after participants spent 20 minutes using MentorSL. Questions in domains (D), (F), and (G) were only asked after participants spent 20 minutes using MentorSL.

### Statistical Analysis

Statistical analysis was performed using SAS (Cary, USA). Data were presented as ratios and percentages. The chi-square test was used for significance testing.

## Results

### Demographic Data

There were 57 participants in total, median age was 28.1 years (range 24-43). There were 32 females (32/57, 56%) and 25 males (25/57, 44%).

Of the total participants, 1/57 (2%) qualified in 2004, 3/57 (5%) qualified in 2007, 23/57 (40%) qualified in 2008, 13/57 (23%) qualified in 2009, and 17/57 (30%) qualified in 2011. Of the total participants, 20/57 (35%) were in foundation year 1 (FY1), 22/57 (39%) were in core surgical training 1 (CT1), and 15/57 (26%) were in core training 2 (CT2).

Of the 57 participants, 40/57 (70%) participants reported that they had firm plans for which specialty they would like to enter, 3/57 (5%) had no plans as yet, and 14/57 (25%) were unsure of their choice. The response rate for the questionnaire was 35/57 (61%).

**Figure 2:**
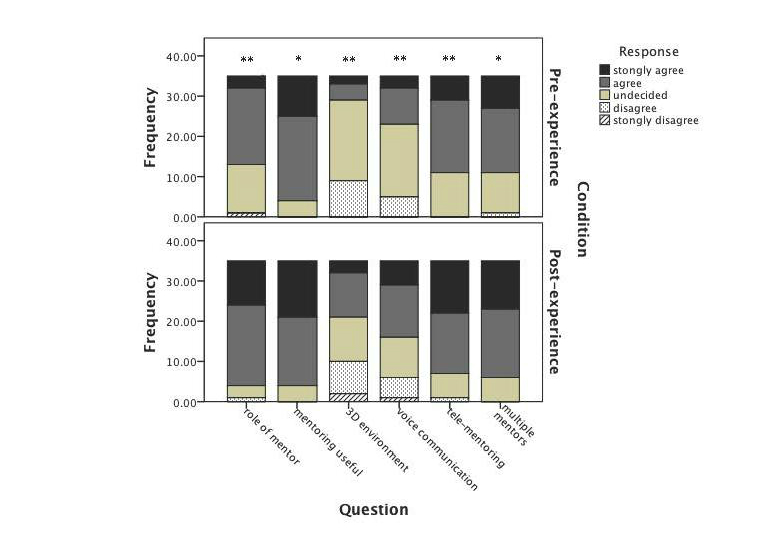
Stacked bar chart of response to questions pre- (above) and post-experience (below) of the MentorSL system. *Improvement in response; *P*<.05, **improvement of response; *P*<.001 (one-way chi-square test).

In terms of previous experience with the 3D virtual environments, 6/57 (11%) had had previous experience and 51/57 (89%) had no experience or were unsure. Mentee perceptions are described below and summarized in Figure 2.

### Perceptions Regarding Concepts of Mentoring

With regards to having understood of the roles of a mentor, prior to the experience, 3/35 (9%) said they strongly agreed, 19/35 (54%) said they agreed, 12/35 (34%) were unsure, and 1/35 (3%) strongly disagreed. Following the experience, there was a statistically significant improvement toward agreement (*P*<.001; chi-square test). Of 35 participants, 11/35 (31%) strongly agreed, 20/35 (57%) agreed, 3/35 (9%) were unsure, and 1/35 (3%) disagreed.

With regards to whether mentoring was thought to be useful, 10/35 (29%) strongly agreed, 21/35 (60%) agreed, and 4/35 (11%) were unsure. Following the experience, there was a statistically significant improvement toward agreement (*P*=.04; chi-square test). Of 35 participants, 14/35 (40%) strongly agreed, 17/35 (49%) agreed, and 4/35 (11%) were unsure.

### Perceptions Regarding Mentoring via the 3D Web

When asked whether they thought whether a 3D virtual environment would be useful in mentoring prior to experiencing it, 2/35 (6%) strongly agreed, 4/35 (11%) agreed, 20/35 (57%) were unsure, and 9/35 (26%) disagreed. Following the experience, there was a statistically significant improvement toward agreement (*P*<.001; chi-square test). Of 35 participants, 3/35 (9%) strongly agreed, 11/35 (31%) agreed, 11/35 (31%) were unsure, 8/35 (23%) disagreed, and 2/35 (6%) strongly disagreed.

### Perceptions Regarding the Practicalities of MentorSL

Prior to experiencing MentorSL, when asked whether voice communication would be useful in the mentoring relationship, 3/35 (9%) strongly agreed, 9/35 (26%) agreed, 18/35 (51%) were unsure, and 5/35 (14%) disagreed. Following experiencing MentorSL, there was a statistically significant improvement toward agreement (*P*<.001; chi-square test). Of 35 participants, 6/35 (17%) strongly agreed, 13/35 (37%) agreed, 10/35 (29%) were unsure, 5/35 (14%) disagreed, and 1/35 (3%) strongly disagreed.

When asked regarding navigation in SL was sufficiently simple to use, 6/35 (17%) strongly agreed, 11/35 (31%) agreed, 14/35 (40%) were undecided, 2/35 (6%) disagreed, and 2/35 (6%) strongly disagreed.

When asked whether the search for mentor facility in MentorSL was sufficiently simple to use, 7/35 (20%) strongly agreed, 6/35 (17%) agreed, 19/35 (54%) were undecided, 2/35 (6%) disagreed, and 1/35(3%) strongly disagreed.

When asked whether the meeting scheduling facility in MentorSL was sufficiently simple to use, 4/35 (11%) strongly agreed, 11/35 (31%) agreed, 18/35(51%) were undecided, and 2/35 (6%) disagreed.

When asked regarding ease of using voice communication in SL, 6/35 (17%) strongly agreed, 10/35 (29%) agreed, and 19/35 (54%) were undecided.

Regarding overall satisfaction with MentorSL, 6/35 (17%) reported very good, 24/35 (69%) reported adequate, 4/35 (11%) reported slightly disappointing, and 1/35 (3%) reported very poor.

### Perceptions Regarding Tele- and Multiple Mentoring

Regarding the usefulness of a specialist mentor who may be geographically remote, prior to the experience, 6/35 (19%) strongly agreed, 18/35 (51%) agreed, and 11/35 (31%) were unsure. Following the experience, there was a statistically significant improvement toward agreement (*P*<.001; chi-square test). Of 35 participants, 13/35 (37%) strongly agreed, 15/35 (43%) agreed, 6/35 (17%) were unsure, and 1/35 (3%) disagreed.

When asked regarding the perceived benefits of having multiple mentors available for specific mentoring needs, prior to the experience, 8/35 (23%) strongly agreed, 16/35 (46%) agreed, 10/35 (29%) were unsure, and 1/35 (3%) disagreed. Following the experience, there was a statistically significant improvement toward agreement (*P*=.002; chi-square test). Of the participants, 12/35 (34%) strongly agreed, 17/35 (49%) agreed, and 6/35 (19%) were unsure.

### Perceptions Regarding Further Enhancement of Virtual Mentoring

When asked whether participants thought that real life facial recognition and animation of avatar facial features would be useful, 2/35 (6%) strongly agreed, 13/35 (37%) agreed, 13/35 (37%) were undecided, and 7/35 (20%) disagreed.

When asked whether hand gesture recognition and animation of avatar would be useful, 2/35 (6%) strongly agreed, 12/35 (34%) agreed, 14/35 (40%) were undecided, and 7/35 (20%) disagreed.

### Perceptions Regarding Future Use of MentorSL

When asked whether participants would use MentorSL in the future, 4/35 (11%) strongly agreed, 15/35 (43%) agreed, 10/35 (29%) were undecided, 4/35 (11%) disagreed, and 2/35 (6%) strongly disagreed.

## Discussion

### Principal Findings

This study demonstrates that doctors in postgraduate surgical training are willing to “buy-in” to a virtual mentoring system in SL. The most well-received facilities were those of tele- and multiple mentoring and that of voice communication. The implication of these findings is that this mentoring system may be able to deliver mentoring to this group of doctors in a manner commensurate with their needs.

The response rate of 61% (35/57) in this study, seemingly low and a limitation of the study, is commensurate with other studies in this population [29]. We would suggest that a subsequent usability study would result in increased participant involvement and perceived benefit. Thus, we would suggest that this would be the lower limit of what a future usability study would engender. This study is not able to inform on the potential benefit of virtual mentoring using this system, and further work will be needed to establish this.

### Perceptions

It the context of team training for triage of mass casualties, it has been demonstrated that trainees quickly adapt to a virtual environment and find it an experience that is beneficial to their professional development [30].

Despite the all-pervasive nature of the Internet in today’s society, 89% (51/57) of participants had no significant previous experience of 3D Web 3.0 technology. Despite this, we found that only 17% (6/35) of participants disagreed or strongly disagreed, following a short introduction, with using the system in the future.

Central to the provision of a virtual “just-in-time” mentoring system is the mentee perceiving the need for being mentored. At outset, only 21/35 (60%) participants agreed or strongly agreed that they understood the concept of mentoring; this improved to 31/35 (89%). In addition, the initial high agreement with the usefulness of mentoring was maintained following the introduction (31 predemonstration vs 32 postdemonstration).

The specific use of the 3D virtual world for mentoring is perhaps the most contentious issue to be assessed in the confines of a short introduction. More formed decisions will most likely require the on-going usage of the system by mentees. This seems to be reflected in that 14/35 (40%) were positive regarding the system, and 11/35 (31%) were unsure.

Importantly, the more immediate and apparent facilitatory benefits of the system seemed to be well received by the participants. This was reflected by the strong performance in the voice communication, tele-, and multiple-mentoring domains. Indeed, the voice communication domain showed a large increase in agreement from 4/35 (11%) to 16/35 (46%). The user-friendliness of the voice communication was found to have a major impact in acceptance of a SL training program for nurses [24].

There may be barriers to the adoption of these new technologies for medical mentoring. Hansen et al recalled Roger’s diffusion of innovation theory in explaining attributes of a new technology affecting an individual’s decision to adopt [31]. These attributes include the relative advantage of the innovation over the idea it supersedes, how the innovation meets the needs of potential adopters, how difficult the innovation is to understand and use, how the innovation may be tested in a timely fashion, and how outcomes associated with the innovation are visible to others. Interestingly, a study investigating these factors in adoption of e-mentoring by Greek mentors reported that only relative advantage was a significant factor in adoption [32]. Other potential drivers for adoption that may be important to further work are alluded to by the “Uses and Gratification” theory [33], suggesting that various forms of gratification affect utility. The well-established “Technology Acceptance Model” emphasizes “perceived usefulness” and “perceived ease-of-use” as important factors in new technology adoption [34], and these also may be important domains to investigate in future work.

With regards to future developments in the MentorSL, equivalent numbers were positive regarding animation of the avatars facial features and hand gestures to improve the experience.

### Conclusions

We have demonstrated that the MentorSL system has the potential to be well accepted by mentees. This may be a reflection of the rapidly acquired understanding of the role of a mentor and the feeling of need for mentoring. Junior surgical trainees are able to rapidly familiarize with this novel communication modality and seem interested in further expansion of the virtual mentoring experience using facial and gesture recognition and avatar animation technology. Further work is required to evaluate utilization of this virtual mentoring facility when made available to doctors in postgraduate surgical training and to establish benefit. We are currently establishing a pilot study to trial medical mentoring using MentorSL in a cohort of surgical trainees in the London Postgraduate School of Surgery.

## Multimedia Appendix 1

Questionnaire.

## Abbreviations

CT1: core surgical training 1
CT2: core surgical training 2
FY1: foundation year 1
FY2: foundation year 2
SL: Second Life
3D: three-dimensional
