# Supplementary material for: Surgical Trainee Opinions in the United Kingdom Regarding a Three-Dimensional Virtual Mentoring Environment (MentorSL) in Second Life: Pilot Study
Source: JMIR Serious Games. 2013 Sep 20;1(1):e2. doi: 10.2196/games.2822 (PMC4307826; doi:10.2196/games.2822)
Supplement: Supplementary file 1 [file games_v1i2e2_app1.pdf]

Questionnaire for Trainees experience of Mentor[SecondLife]

A) Demographic data

1. 1. Gender: Male/ Female

2. Age in years

3. Year of qualification

4. Grade
   FY1 FY2 CT1 CT2 ST1/ 2/ 3/ 4/ 5/ 6 Locum appointment; Other

5. Do you have any previous experience with the 3D web?

   Strongly agree; Agree; Unsure; Disagree; Strongly disagree

6. Prior to attending, did you have any plans regarding which specialty/ subspecialty you would like to practice?

   Strongly agree; Agree; Unsure; Disagree; Strongly disagree

B) Regarding the concept of mentoring

1. Are you clear on the responsibilities and roles of a mentor?

   Yes; No; Unsure

2. Do you think mentoring is of use to you in your future training?

   Strongly agree; Agree; Unsure; Disagree; Strongly disagree

C) Regarding the concept of mentoring via the 3D web

1. Do you find the experience of mentoring in a 3D interactive environment to be useful?

   Strongly agree; Agree; Unsure; Disagree; Strongly disagree

2. Do you find the use of voice communication in the 3D environment to be of potential benefit?

   Strongly agree; Agree; Unsure; Disagree; Strongly disagree

D) Regarding the practicalities of mentor[second life]

1. Did you find navigating within SecondLife sufficiently simple to use?

   Strongly agree; Agree; Unsure; Disagree; Strongly disagree

2. Did you find the search facility within the Mentor[SecondLife] environment sufficiently simple to use?

   Strongly agree; Agree; Unsure; Disagree; Strongly disagree

3. Did you find the meeting scheduling facility within the Mentor[SecondLife] environment sufficiently simple to use?

Strongly agree; Agree; Unsure; Disagree; Strongly disagree

4. Did you find the voice communication within the Mentor[SecondLife] environment sufficiently simple to use?

   Strongly agree; Agree; Unsure; Disagree; Strongly disagree

5. How satisfied overall with the Mentor[SecondLife] environment were you?

   Very good; Adequate; Slightly disappointing; Very Poor

E) Regarding the utility of tele- and multiple mentoring

1. Does the availability of mentors with particular expertise in certain aspects of mentoring who may be geographically remote as a useful facility?

   Strongly agree; Agree; Unsure; Disagree; Strongly disagree

2. Does the availability of multiple mentors with expertise in various aspects of mentoring who may be geographically remote as a useful facility?

   Strongly agree; Agree; Unsure; Disagree; Strongly disagree

F) Regarding further enhancement of the virtual mentoring experience

1. Do you think facial recognition and projection onto your and your mentors avatar would enhance the mentoring experience?

   Strongly agree; Agree; Unsure; Disagree; Strongly disagree

2. Do you think gesture recognition and projection onto your and your mentors avatar would enhance the mentoring experience?

   Strongly agree; Agree; Unsure; Disagree; Strongly disagree

G) Regarding future use of Mentor[SecondLife]

1. Would you be interested in utilising this facility in the future?

Strongly agree; Agree; Unsure; Disagree; Strongly disagree
